# Physical, Thermal, and Antibacterial Effects of Active Essential Oils with Potential for Biomedical Applications Loaded onto Cellulose Acetate/Polycaprolactone Wet-Spun Microfibers

**DOI:** 10.3390/biom10081129

**Published:** 2020-07-31

**Authors:** Helena P. Felgueiras, Natália C. Homem, Marta A. Teixeira, Ana R. M. Ribeiro, Joana C. Antunes, Maria Teresa P. Amorim

**Affiliations:** Campus of Azurém, Centre for Textile Science and Technology (2C2T), Department of Textile Engineering, University of Minho, 4800-058 Guimarães, Portugal; natalia.homem@2c2t.uminho.pt (N.C.H.); martaalbertinateixeira@gmail.com (M.A.T.); rita.ribeiro_02@hotmail.com (A.R.M.R.); joana.antunes@2c2t.uminho.pt (J.C.A.); mtamorim@det.uminho.pt (M.T.P.A.)

**Keywords:** cinnamon leaf oil, cajeput oil, clove oil, antibacterial properties, microfibers immobilization

## Abstract

New approaches to deal with the growing concern associated with antibiotic-resistant bacteria are emerging daily. Essential oils (EOs) are natural antimicrobial substances with great potential to mitigate this situation. However, their volatile nature, in their liquid-free form, has restricted their generalized application in biomedicine. Here, we propose the use of cellulose acetate (CA)/polycaprolactone (PCL) wet-spun fibers as potential delivery platforms of selected EOs to fight infections caused by *Staphylococcus aureus* (*S. aureus*) and *Escherichia coli* (*E. coli*). Twenty EOs were selected and screened for their minimal inhibitory concentration (MIC), using the antibiotic ampicillin as positive control. The cinnamon leaf oil (CLO), cajeput oil (CJO), and the clove oil (CO) were the most effective EOs, against the Gram-positive (MIC < 22.38 mg/mL) and the Gram-negative (MIC < 11.19 mg/mL) bacteria. Uniform microfibers were successfully wet-spun from CA/PCL with an averaged diameter of 53.9 ± 4.5 µm, and then modified by immersion with CLO, CJO and CO at 2 × MIC value. EOs incorporation was confirmed by UV-visible spectroscopy, Fourier-transformed infrared spectroscopy, and thermal gravimetric analysis. However, while microfibers contained ampicillin at MIC (control) after the 72 h modification, the CLO, CO and CJO-loaded fibers registered ≈ 14%, 66%, and 76% of their MIC value, respectively. Data showed that even at small amounts the EO-modified microfibers were effective against the tested bacteria, both by killing bacteria more quickly or by disrupting more easily their cytoplasmic membrane than ampicillin. Considering the amount immobilized, CLO-modified fibers were deemed the most effective from the EOs group. These results indicate that CA/PCL microfibers loaded with EOs can be easily produced with increased antibacterial action, envisioning their use as scaffolding materials for the treatment of infections.

## 1. Introduction

For many years, biomolecules have been used in biomedical applications to fight infections and/or instigate specific cell/tissue responses. A wide range of antimicrobial agents fall within the category of biomolecules, including the antibiotics, peptides, enzymes, organic nanoparticles, and natural extracts [1]. From those, the most widely used are the antibiotics. However, their excessive consumption and misuse have accelerated the rise of antibiotic-resistant microorganisms, which, nowadays, constitute one of the dominant challenges to global health [2]. In fact, recent projections indicate that bacterial infections may be the cause of approximately 10 million annual deaths worldwide by 2050, a number considerably superior to current cancer statistics [3]. Therefore, alternative strategies to antibiotics are in urgent need to treat infections. Natural products derived from plants with antimicrobial, anti-inflammatory, antioxidant, and chemo-preventive properties have been used for many generations in traditional medicine [4]. Nowadays, they are gaining new status as an alternative to antibiotics, by preventing and treating infectious diseases with little impact in the environment [5]. Essential oils (EOs) are mixtures of aromatic, volatile, lipophilic biomolecules, extracted from regions of plants (e.g., flowers, leaves, twigs, bark, wood, fruits, etc.) in which they work as secondary metabolites, defending the host from microbial invasion [1,5]. They are formed of complex mixtures of hydrophobic molecules, including thymol, carvacrol, and eugenol (among others), which exhibit a broad spectrum of antimicrobial activity against bacteria, fungi, and viruses. Research has also demonstrated the EOs potential to replace antibiotics due to their inherent and strong anti-inflammatory, antiseptic, analgesic, spasmolytic, anesthetic, and antioxidative properties [6]. Still their cytotoxicity at increased concentrations, their low resistance to degradation by external factors (e.g., temperature, light, moisture), and their volatility in their free, liquid form remain difficult challenges to overcome for their expanded use. Here, fibers and fibrous constructs have emerged as optional platforms for their targeted and therapeutic delivery, this way promoting their application in the combat against drug-resistant bacteria [7,8].

The wet-spinning technique is a nonsolvent induced phase inversion approach that allows the production of continuous polymeric microfibers, with uniform morphology, by injecting a polymer solution into a nonsolvent coagulation bath that prompts the solidification of the extruded material [7,9]. This technique has been raising attention in the biomedical field for its simple set-up, enabling the production of fibers at the microscale for controlled drug release and/or tissue engineering applications. Further, wet-spinning allows 3D constructs to be formed with an intricate fibrous network that facilitates cell infiltration within the scaffold’s inner regions, something that is limited in electrospun nanofibrous meshes due to their small pore sizes and high fiber packing density [10]. This technique was originally introduced in biomedicine to process natural polymers, such as chitosan, collagen, or silk fibroin, which were susceptible to degradation when subjected to other conventional spinning techniques or very difficult to establish optimal processing parameters via electrospinning [11]. The possibility of easily loading wet-spun fibers with a wide range of therapeutic agents has been very important to broaden its use to synthetic polymers [12].

In this study, we propose the production of cellulose acetate (CA) and polycaprolactone (PCL) microfibers loaded with selected EOs for potential applications in the biomedical field. CA is the acetate ester of cellulose and one of its most common derivatives. CA is a bio-based polymer, endowed with a unique nanostructure and remarkable physicochemical properties, is biocompatible and biodegradable, and can be efficiently processed into membranes, films, and fibers [13,14,15,16]. It is also capable of high entrapment efficiency and, consequent, delivery of diverse therapeutic drugs. Furthermore, it has been shown that these properties can be enhanced by blending it with other polymers, such as PCL [17]. This synthetic polymer displays excellent mechanical performance, miscibility with other polymer solutions, and is also biocompatible and biodegradable [18]. To the authors’ knowledge, this is the first report on the effect of EOs on the physical, thermal, and antimicrobial properties of CA/PCL microfibers, processed by wet-spinning. Here, various EOs were examined and the most effective against Gram-positive and Gram-negative bacteria were immobilized onto the biodegradable microfibers and further explored for their effects on the fiber’s physical, thermal, and antimicrobial properties.

## 2. Materials and Methods

### 2.1. Materials

EOs were purchased from Folha d’Água Company (Portugal) and are listed in Table 1. Trypticase soy broth (TSB) and trypticase soy agar (TSA) were acquired from VWR, while Mueller Hinton broth (MHB) was obtained from CondaLab. Bacteria were supplied from American Type Culture Collection (ATCC), encompassing Gram-positive bacteria, *Staphylococcus aureus* (*S. aureus*, ATCC 6538), and Gram-negative bacteria, *Escherichia coli* (*E. coli*, ATCC 25922). The antibiotic ampicillin was purchased from Sigma and used as positive control in all the experiments. 

Cellulose acetate (CA, Mn = 30,000 and 39.8 wt % acetyl content) and polycaprolactone (PCL, Mn = 45,000) were purchased from Sigma and used in the production of wet-spun microfibers. Acetone (Ace, Millipore) and glacial acetic acid (AA, Fisher Scientific) were used as polymer solvents, and absolute ethanol (EtOH, Millipore) was employed as coagulation bath.

EOs were diluted in MHB at 40–0.078%, which corresponded to an average maximum concentration of 385.442 ± 56.877 mg/mL and an average minimum concentration of 0.753 ± 0.111 mg/mL; maximum and minimum concentrations for each oil were dependent on their inherent density (Table 1). Ampicillin (A) was prepared in distilled water (dH_2_O) at concentrations 1000–1.95 µg/mL. For the purpose of this work, fiber samples were labeled with an “F” prior to the name of the immobilized biomolecule.

### 2.2. Agar-Well Diffusion Assay

The EOs antibacterial activity was initially assessed visually against *S. aureus* and *E. coli* via the Kirby-Bauer method. Briefly, bacteria inoculums were prepared in TSB and left to grow overnight at 37 °C and 120 rpm. Then, their concentration was adjusted to 1.0 × 10^7^ colony forming units (CFUs)/mL and 1 mL was collected and combined with 14 mL of TSA warmed at approximately 45 °C. The 15 mL bacterial solution was poured into 90 mm diameter Petri dishes and left to solidify. Sterilized punchers were used to generate 6 mm diameter holes on the agar. A 40 µL volume of each antimicrobial agent, at the highest concentration studied (40%), were introduced in the respective holes. Plates were incubated at 37 °C for 24 h. Zones of inhibition (ZoI) were observed and measured to confirm the EOs’ antibacterial efficacy.

### 2.3. Minimum Inhibitory Concentrations (MICs)

MICs were determined using the broth microdilution procedure described by Wiegand et al. [19], which adapts the standard published by the Clinical and Laboratory Standards Institute (CLSI) and the European Committee on Antimicrobial Susceptibility Testing (EUCAST) [20].

Working solutions were prepared as described in Section 2.1, and added to the first column of 96-well plates in a volume of 100 μL. Serial dilutions (1:2) were made with MHB in the consecutive wells, to a final volume of 50 μL. Then, to each of these wells, 50 μL of the bacteria suspensions prepared at 1 × 10^7^ CFUs/mL in MHB were added. Free bacteria suspensions and culture media were used as controls. Absorbance readings at a wavelength of 600 nm (EZ READ 2000 Microplate Reader, Biochrom Ltd., Cambridge, UK) were made before and after plate incubation for 24 h at 37 °C and 120 rpm. The MIC value for each agent/bacteria combination was established as the concentration at which bacteria did not show any growth, determined visually, and confirmed by the differences in absorbance readings. The existence of viable cells at MIC and at concentrations in its vicinity (concentration higher and lower than MIC value) was determined by measuring the number of CFU s/mL. Briefly, aliquots of 10 µL of each cell suspension, diluted from 10^1^ to 10^5^ in phosphate buffer saline solution (PBS), were cultured in TSA plates for 24  h at 37 °C, and colonies were counted. The three most effective EOs against both bacteria (lowest MICs) were highlighted from the group and used in the subsequent experiments.

### 2.4. Cell Wall Disruption: Scanning Electron Microscopy (SEM) Observations

To analyze the EOs capacity to interfere with the cell morphology of Gram-positive and Gram-negative bacteria, visual studies resorting to SEM were conducted. Here, only the most effective EO was selected since the mechanisms of action are very similar among EOs [5]. Bacteria suspensions were prepared at 1 × 10^5^ CFUs/mL in MHB and combined at 50% (v/v) with the selected EO (cinnamon leaf oil (CLO)) at MIC value. A 500 µL volume of each solution (250 µL CLO + 250 µL bacteria) were left in direct contact with 12-well tissue culture plates (TCPS) and incubated at 37 °C for 24 h at 120 rpm. Untreated bacteria were used as control. Afterwards, culture media was removed and 500 µL of 2.5% (v/v) glutaraldehyde in PBS were added to each sample for 1 h at room temperature (RT), to promote cell fixation to the TCPS wells. Plates were gently rinsed with dH_2_O and submitted to a dehydration process using serial ethanol dilutions, 55%, 70%, 80%, 90%, 95%, and 100% (v/v), each solution was left in the TCPS for 30 min at RT, and then carefully discarded. After the last solution, the remaining ethanol was evaporated at RT. Discs were cut from the TCPS wells, containing the fixated and dehydrated bacteria, using a hot press-on apparatus and covered with a thin film (10 nm) of Au-Pd (80–20 wt %) in a 208HR high-resolution sputter coater (Cressington Company, Watford, UK) coupled to an MTM-20 Cressington high-resolution thickness controller. Cell morphology was observed via FEG-SEM (NOVA 200 Nano SEM, FEI Company, Hillsboro, Oregon, USA), using an electron accelerating voltage of 10 kV.

### 2.5. Wet-Spun Fiber Production and EO Immobilization

CA/PCL spinning solution was prepared by dissolving CA at 10% w/v in AA and PCL at 14% w/v in 3:7 v/v Ace/AA. The ratio of blended polymer (CA/PCL) was 3:1 v/v. These concentrations and ratios were selected based on previously published work [17]. The complete dissolution of polymers was achieved under stirring at 90 °C, for 1 h (300 rpm), followed by overnight slow stirring (30 rpm) to remove existing air bubbles. CA/PCL was then processed by wet-spinning (Figure S1). The set-up was composed of a syringe pump (NE-1600, New Era Pump Systems, Norleq, Porto, Portugal) coupled to a 20 mL syringe and 18-gauge (G) needle. The polymer solution was extruded at a flow rate of 0.5 mL/h within an EtOH coagulation bath, at ≈21.5 °C. The distance between the needle tip and the bottom of the coagulation bath was kept constant (≈3 cm). Microfibers were collected manually and stored in the form of 21 cm length samples (ca. 0.5 mg) for subsequent testing.

Microfibers were divided into five groups: (1) unloaded (control) and loaded with (2) ampicillin (positive control), (3) CLO, (4) CO, and (5) CJO. Fibers were immersed in an ethanol solution containing each of the EOs at a concentration of 2 x MIC for 72 h at RT and 200 rpm, for immobilization. This period was selected based on the control sample containing ampicillin, which reached maximum incorporation (equivalent to MIC) at this moment, after measurements at 24 and 48 h being insufficient (data not shown); longer periods would imply an excess of ampicillin at the fiber’s surface. Peaks of absorbance (207–212 nm) were established for each oil by conducting calibration curves that related absorbance to EOs concentration. The percentage of EO incorporated was determined by measuring the difference of absorbance at times 0 and 72 h (UV–VIS 1800, Shimadzu, Shimadzu Schweiz GmbH, Reinach BL, Switzerland). Any alterations in the absorbance of the control samples (unloaded) were considered and subtracted from the final absorbance for each sample.

### 2.6. Fiber Characterization

#### 2.6.1. Morphology: Brightfield Microscopy

Brightfield images of the microfibers morphology were collected using an inverted microscope Leica DM IL LED (Leica Microsystems, Wetzlar, Germany), both in the absence and presence of immobilized antimicrobial agents. After the 72 h immobilization process, samples were washed with dH_2_O for 5 min (3 times), mounted with VECTASHIELD^®^ Mounting Medium (Vector Laboratories, Burlingame, California, USA) and sealed for observation. Images were captured with 10× and 40× objectives, with the averaged fiber diameter being determined from 5 images collected for each sample type and measured through ImageJ software (version 1.52a).

#### 2.6.2. Chemical Composition: Attenuated Total Reflectance with Fourier-Transform Infrared Spectroscopy (ATR-FTIR)

An IRAffinity-1S, SHIMADZU spectrophotometer (Kyoto, Japan), with an ATR accessory (diamond crystal) was used to record the CA/PCL fibers ATR-FTIR spectra. For each fiber, a total of 45 scans were performed at a spectral resolution of 8 cm^−1^, over the wavenumber range of 400–4000 cm^−1^.

#### 2.6.3. Thermal Stability: Thermal Gravimetric Analysis (TGA)

TGA measurements were conducted on a STA 7200 Hitachi^®^ (Fukuoka, Japan) using a platinum pan. The TGA trace was obtained in the range of 25–600 °C under a dynamic nitrogen atmosphere, flow rate of 200 mL/min and temperature rise of 10 °C/min. Results were plotted as a percentage of mass loss vs. temperature and as weight loss rate vs. temperature (derivative thermogravimetric curve, DTG).

### 2.7. Time-Kill Kinetics

Bacteria suspensions were prepared at 1 × 10^5^ CFUs/mL in MHB and combined with the antimicrobial agents at MIC percentage (according to the EOs loading efficiency results) or the modified fibers. Control groups were prepared without the addition of any agent or by using unmodified fibers. Bacteria-containing solutions were incubated at 37 °C and 120 rpm. After 0 (before action), 1, 2, 4, 6, and 24 h of incubation, bacteria were serially diluted (10^1^ to 10^5^ in PBS), cultured on TSA plates, and further incubated for another 24 h at 37 °C. Colonies of surviving bacteria were counted and reported as mean ± standard deviation (S.D.). Log reduction determinations were also conducted between bacteria solutions with and without antimicrobial agents and fibers unloaded and loaded and reported as mean ± S.D. Data were collected in triplicate and processed using the GraphPad Prism 7.0 software.

### 2.8. Membrane Permeability

The bacteria membrane permeability was evaluated by adapting the procedure described by Kong et al. [21]. Differences in relative electric conductivities (REC) of bacteria suspensions by the addition of antimicrobial agents can be used to determine their capacity to penetrate the membrane, as conductance has been suggested as an indicator not only of pore size but also interaction of permeating ions with wall channels in various bacteria [22]. Here, REC were measured after subculturing *S. aureus* and *E. coli* bacteria in MHB overnight. Bacteria were collected by centrifugation at 5000 rpm for 10 min, their concentration adjusted to 1 × 10^5^ CFUs/mL, and then washed in a 5% glucose solution until their electric conductivities were close to that of 5% glucose (isotonic bacteria). The EOs (at a loaded concentration on microfibers) and microfibers (unloaded and loaded) were then added to the 5% glucose solution and electric conductivities of the mixtures were marked as L1. Ampicillin, EOs, and microfibers (unloaded and loaded) were added into the isotonic bacteria solution. After completely mixed, the samples were incubated at 37 °C for 6 h, and their conductivities measured and marked as L2. The control was the bacteria in 5% glucose treated in boiling water for 5 min, marked as L0. The bacterial cell membrane permeability was expressed as the ratio of REC, which was calculated as:

(1) $$REC\left(\%\right)=\frac{\left(L2-L1\right)}{L1}x100$$

Data were collected in triplicate and reported as mean ± S.D. using the GraphPad Prism 7.0 software.

## 3. Results and Discussion

### 3.1. Agar-Well Diffusion

EOs were initially screened for their antibacterial activity against the Gram-positive bacteria *S. aureus* and the Gram-negative bacteria *E. coli* via the agar-well diffusion test. Here, the maximum tested concentrations were employed (40%). Table 2 gives a first look on the antibacterial action of the 20 selected EOs, which can be classified following the Rota et al. scale in weak if ZoI ≤ 12 mm, moderate if ZoI ranged between >12 and <20 mm, and strong if ZoI ≥ 20 mm [23].

From all tested agents, ampicillin formed the strongest ZoI (Table S1). Ampicillin is a semi-synthetic penicillin-like molecule that acquires its antimicrobial properties from the presence of a β-lactam ring (four-membered cyclic amide) in its structure, which is known to affect the growth, viability, shape division and integrity of bacterial cells. It is useful in the treatment of infections mediated by both Gram-positive and Gram-negative bacteria [24]. Even though its antibacterial potential is well-known, the results from Table 2 are not a statement of its superior antimicrobial contribution but are rather associated with its ability to diffuse along the agar. The agar tortuosity may hinder the EOs diffusion, particularly those with a superior viscosity and density (Table 1). As such, the results from Table 2 can only be considered as indicatives of the antimicrobial potential of the agents and not of their intensity. Still, when looking at both the *S. aureus* and *E. coli* data, it is possible to highlight the CJO, TTO, SO, CLO, NO, and CO as the most probable to present a superior antimicrobial action as their ZoI falls within the moderate activity range for both cases. Data also demonstrates the increased effectiveness of the EOs against the Gram-positive bacterium compared to the Gram-negative. These results can be explained by the differences in the cell wall structure of these bacteria, since the absence of an outer cell membrane and periplasm in the Gram-positive bacteria allows higher molecules permeability, while the Gram-negative are capable of restricting diffusion of hydrophobic molecules through its lipopolysaccharide envelope reducing penetration [25,26]. From the group, the HCO, SAO, and WO did not form any ZoI even though reports have been published on their antibacterial action [27,28,29]. This supports the premise established by Tavares et al. that the plant origin and the extraction methods of natural products employed by each company may influence the properties of the final product [5].

### 3.2. MICs

MICs were established for each bacterium (Table 3), revealing the strongest and weakest antimicrobial agents from the tested group. Apart from HCO, SAO, and WO, which results attest to the absence of the ZoI (Table 2), all agents were found active against one and/or two microorganisms. In the case of HCO, SAO, and WO a larger concentration than the maximum tested would be required.

As anticipated, the ZoI results did not reflect the reality of the quantitative measurements. Ampicillin was very effective against both bacteria, with an average MIC of ≈31.3 µg/mL; however, between the EOs, the CLO, CO, CJO, and TTO were the most effective against the bacteria even though their ZoI were not the largest (Table 2 and Table S1).

Data established the CLO, CO, and CJO as the most antibacterial from the EOs group, with the last being closely followed by TTO. Both CJO and TTO belong to the *Melaleuca* genus, sharing many identical components: CJO encompasses 1,8-cineole (42%), α-terpineole (18%), caryophyllene (11%), limonene (4%), α-pinene (3%), and eugenol (1%) (v/v) [30], while TTO comprises terpinen-4-ol (40%), γ-terpinene (20%), α-terpinene (10%), 1,8-cineole (5%), α-pinene (3%), and limonene (1%) (v/v) [31]. Because of the similarities between CJO and TTO only one of the oils was selected for further study. The TTO was excluded due to its relatively higher MIC. All of these components are known to contribute significantly to the EOs antimicrobial activity. The differences in percentage between the identical elements and the abundance of some components in detriment of others may explain the small disparities in the MIC values; for instance, the presence of eugenol in the CJO. Eugenol is characterized by a high antimicrobial action against a variety of microorganisms and ascribes its activity to the presence of free hydroxyl groups capable of binding to proteins, this way preventing enzyme action. Moreover, eugenol is also capable of disrupting the cytoplasmatic membrane increasing the bacterial membrane nonspecific permeability and may affect the transport of ions and adenosine triphosphate (ATP) [32]. The high eugenol content within the CLO and CO, ranging 80%, may also be the reason for the reduced MIC values obtained, ≈0.8 mg/mL. Aside from eugenol, CLO also contains β-caryophyllene (4%), benzyl benzoate (4%), cinnamaldehyde (3%), linalool (2%), and α-terpinene (1%) (v/v) in its composition [33], while CO is also formed by eugenyl acetate (9%) and β-caryophyllene (8%) [34]. These components have all also been reported in the literature as antimicrobial [35].

As observed in the results from Table 2, here too the differences in the cell wall structure of the tested bacterial species may have hindered the EOs action against the Gram-negative bacteria. Indeed, data (Table 3) confirms the enhanced antibacterial effect of many oils against *S. aureus* compared to *E. coli*. From the group of the least effective EOs, the exceptions are the SO and the GO. This demonstrates the potential of these two EOs in fighting infections caused by this Gram-negative bacterium.

### 3.3. Cell Wall Disruption

**Figure 1:**
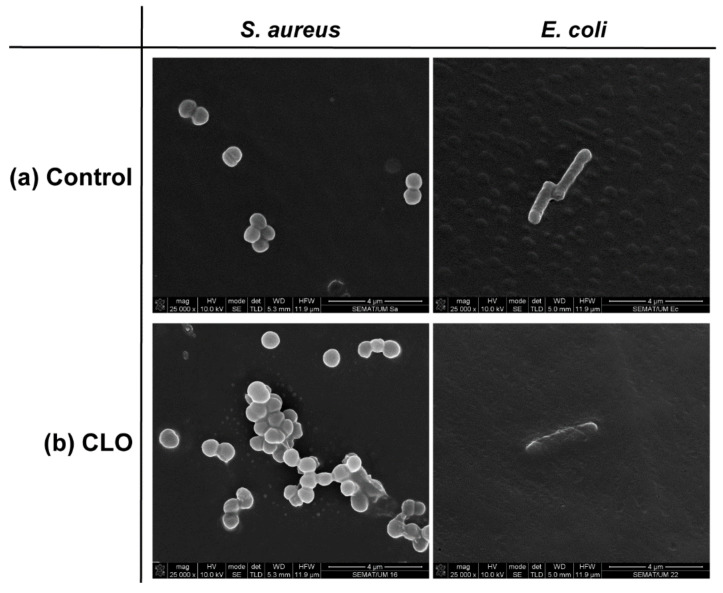
Representative micrographs of *S. aureus* and *E. coli* bacteria morphology in the absence (control, C) and presence of cinnamon leaf oil (CLO), at MIC value.

Considering the similarities between the EOs mechanisms of action against bacteria [5], only the most effective, the CLO, was selected to examine the EOs interference in the cell membrane via SEM. Figure 1 shows the morphology of the bacteria before and after contact with CLO at MIC. Before contact, both bacteria exhibited a smooth, uninterrupted surface. *S. aureus* displayed a coccoid-shaped conformation with grape-like arrangements being very frequent. On its turn, a rod-shaped architecture was observed for *E. coli*, in which the formation of clusters was less common but proliferative signals were evident. In both cases, observations were consistent with the literature [36,37].

EOs act primarily against the cell cytoplasmic membrane by accumulating at its surface and disturbing its structure and functionality. This increases the membrane permeability and the probability of intracellular content leakage, which ultimately leads to cell death [25]. In both bacteria, CLO was capable of increasing the cell permeability, by distorting the membrane appearance and generating holes or wrinkles. Deformed shapes followed by cell shrinkage and blebbing-like architectures were observed in the *S. aureus*. Here too, leakage of cell content by cell membrane rupture was easily detected. In the case of *E. coli*, it appears that the EO surrounded the cells, isolating them. As Gram-negative bacteria are more resistant to hydrophobic biomolecules, to overcome their impermeability, EOs rely on the organism’s isolation to slowly traverse through the outer wall porins [5,25]. In this specific case, CLO may have relied on the abilities of its major component, eugenol, to affect the cells’ intracellular functions or ion transport [32] to progressively kill the bacterial cells without requiring cell content leakage.

### 3.4. Wet-Spun Fibers Modification and Characterization

#### 3.4.1. EO Loading Efficiency

The modification of the wet-spun fibers with the most effective essential oils (smaller MICs), the CLO, CO, and CJO, was accomplished by adsorption via prolonged microfiber exposure (immersion), under constant gentle orbital shaking. The immobilization period was set with ampicillin (positive control) as absorbed biomolecule, in which absorbance measurements were conducted every 24 h of incubation. Here, aliquots of the solution encasing the fibers were recovered and the amount adsorbed by the fibers was determined by the differences in the absorbance between time 0 h and the chosen periods for readings. This adsorption method was selected based on its simplicity and on the presence of free hydroxyl groups (-OH) along the fiber’s surface, which, under prolonged exposure, could form hydrogen bonds with some of the components present on the EOs structure. In this situation, this adsorption mechanism could be further categorized as physisorption. By it, a quicker release of the biomolecules under physiological conditions (desorption) could be attained [8].

Even though solutions were prepared at 2 x MIC, data were here calculated and presented as a percentage of MIC. It was seen that after 72 h of contact, ≈ 106.37% of ampicillin was loaded onto the fibers, which represented ≈33.24 µg/mL, an amount very similar to its MIC value. These results established the maximum exposure time of the microfibers to all the remainder agents. Data reported microfiber loading of ≈14.42% for CLO, which corresponded to ≈0.12 mg/mL, of ≈66.08% for CO or ≈0.55 mg/mL, and of ≈76.48% for CJO or ≈17.12 mg/mL. The differences in the adsorption values may be explained by the location of their available functional groups for binding [38]. Even though many of the CLO, CO, and CJO components are similar, their organization within the biomolecule chemical structure may differ, facilitating or limiting intermolecular bonds with the CA/PCL.

#### 3.4.2. Morphology

**Figure 2:**
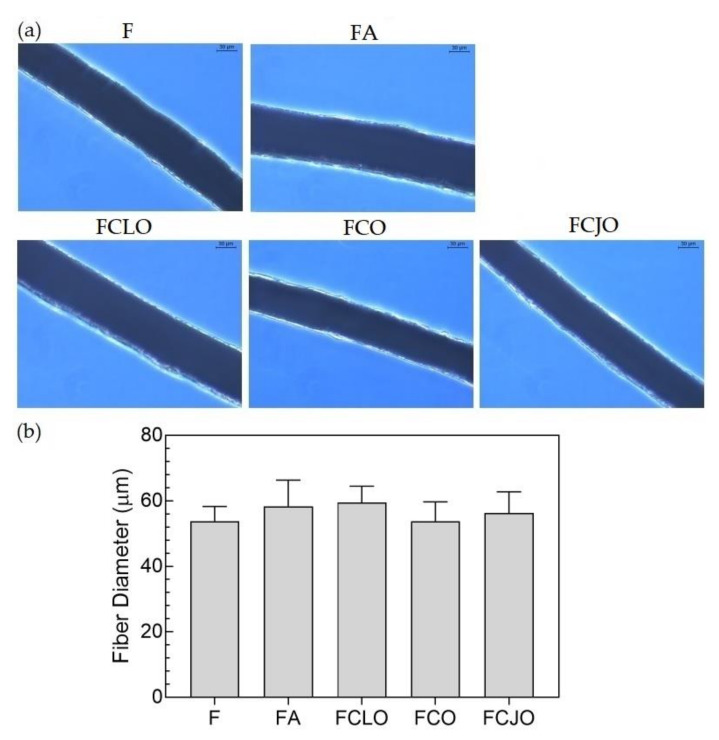
(**a**) Images of the fiber’s morphology unloaded (control) and loaded with antimicrobial agents, captured at 40x magnification using a brightfield microscope (scale bar = 30 µm). (**b**) Distribution of fiber diameters before (C or control) and after the incorporation of the antimicrobial agents, ampicillin, CLO, clove oil (CO), and cajeput oil (CJO). Data derive from measurements of 5 images collected at 10x magnification. There were no statistically significant differences between the unmodified and the modified microfibers.

Microfibers were processed by wet-spinning from a polymeric blend of CA/PCL. Processing conditions were optimized so that the fiber production was continuous and uniform, despite using a manual fiber collector. Figure 2a shows the resulting fiber morphology acquired by brightfield microscopy. The overall homogeneity of the fibers was confirmed even though very small defects (e.g., minor slope in the upper level of the ampicillin fiber), neglectable in light of the average size of each sample (21 cm in length, with ≈0.5 mg in weight), were also observed. Beads or knots were not detected. The fiber’s diameter distribution (Figure 2b) was calculated from 40 measurements made along a group of microfibers treated in equal conditions. The average diameters ranged between 54 and 59 µm: with the control (F) at 53.9 ± 4.5 µm; ampicillin (FA) at 58.4 ± 8.0 µm; FCLO at 59.4 ± 5.0 µm; FCO at 53.8 ± 5.9 µm; and FCJO at 56.3 ± 6.5 µm. Even though the control and the CO-treated samples displayed the smallest diameters from the group, no significant differences were detected between microfibers. This is to be expected since the amount of antimicrobial agent immobilized was very reduced (Section 3.4.1). The behavior of CO compared to CLO or CJO may be explained by the affinity of its components to the CA/PCL polymer blend. CO is composed of 80% eugenol and 9% eugenyl acetate, which structure is very similar to eugenol [34,39]. Both possess free hydroxyl groups that may become available to the polymeric blend for binding, promoting the formation of intricated bonds that may turn the overall surface more hydrophobic, thus reducing the microfiber diameter. To date there are no reports on the affinity of these components to the polymers in study; as such, further studies are required to assess the validity of our premise.

#### 3.4.3. Chemical Composition

**Figure 3:**
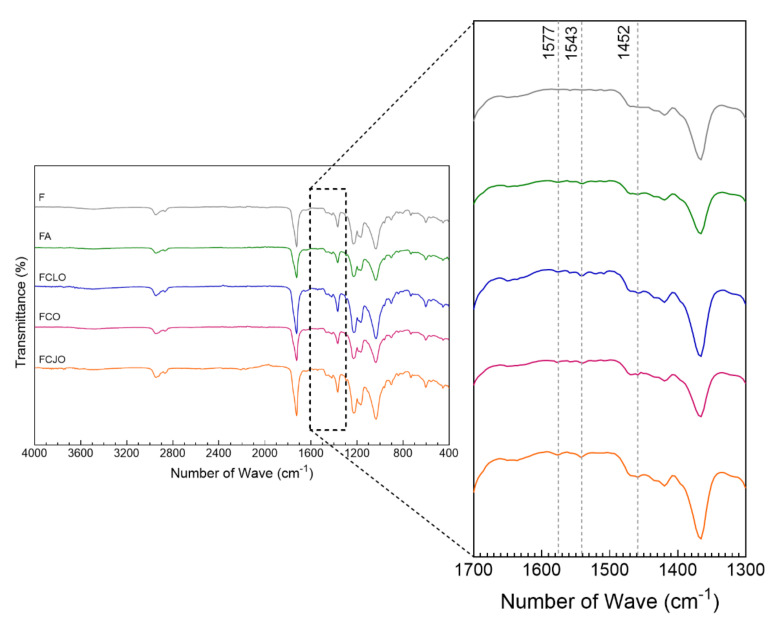
ATR-FTIR spectra of the cellulose acetate (CA)/polycaprolactone (PCL) microfibers unmodified (F) and modified with ampicillin, CLO, CO, and CJO (4000–400 cm^−1^). As the most significant for this study, the section between 1700–1300 cm^−1^ was amplified for a clearer detection of the peak’s characteristic of the immobilized biomolecules.

Spectra of the CA/PCL microfibers, unmodified, and modified with ampicillin, CLO, CO, and CJO, were collected (Figure 3). A small but wide adsorption peak at approximately 3400 cm^−1^ was observed on all fibers. It corresponded to the stretching vibrations of intermolecular hydrogen bonds of the -OH groups and may be associated with the exposure of the fibers to the air humidity. It may also be allocated to the available –OH groups of CA that remained after contact with the EOs. The adsorption peaks observed between 2950–2900 cm^−1^ corresponded to the stretching vibrations of the –CH of methyl groups (–CH_3_) in CA and to the CH_2_ asymmetric stretching vibrations in PCL [14,40]. At 2865 cm^−1^, another peak was detectable and was attributed to the symmetric CH_2_ stretching vibrations also in PCL [40]. At 1735 cm^−1^, a very pronounced peak was detected; this was assigned to the carbonyl (C=O) stretching vibrations and could be allocated to both CA and PCL. At 1370 cm^−1^ an important peak was detected and assigned to –C–H symmetric deformation vibration of the acetate group in CA, and at 1293 cm^−1^ the C–O and C–C stretching vibrations associated with the crystalline phase of PCL were detected [14,40]. Neither of these peaks is usually present on the other polymer, thus confirming their homogenous blend. At 1240 cm^−1^, it was possible to identify the characteristic peak of –C–O–C, which is related to asymmetric stretching vibrations of PCL and/or the CA ester group. The PCL symmetric –C–O–C stretching vibrations were then detected at 1170 cm^−1^ [40]. The presence of CA within the blend was also detected by the band between 1090 and 1000 cm^−1^ which was associated with the stretching modes of –C–O single bonds, and the absorption peak at 904 cm^−1^ which may be due to the combination of the –C–O stretching with the -CH_2_ rocking vibrations [14]. Because of the overlapping of bands between CA and PCL, analogous spectra predominated, and was very difficult to determine the specific contribution of each polymer. Still, the presence of the two polymers was confirmed.

Regarding the modification of the fibers with the antimicrobial agents, the most important observations were made at the absorption peaks of 1577, 1543, and 1452 cm^−1^. Even though small, these were not present on the unmodified microfibers. The peaks at 1577 and 1543 cm^−1^ have been assigned to the aromatic ring C=C skeleton vibration of an aromatic substance. Here, even though the 1577 cm^−1^ is the most commonly found and noticeable in many reports, there have been those that demonstrate the presence of second peaks or even shifts detectable at smaller wavenumbers, including at 1543 cm^−1^ [41]. It may also be explained by the possible interactions made with the polymers CA and PCL by means of hydrogen bonding [42]. These peaks were also identified in the ampicillin-modified fibers and were attributed to inplane vibrations of the –CH groups [δCH(ph)] and to the stretching vibration of the –C–C groups, respectively [43]. At 1452 cm^−1^, the characteristic peak of the –C–OH absorption bending vibration from the alcohol moieties of the EOs was detected. This peak has been identified in all the tested EOs [44,45,46]. The collected data is proof of the presence of the antimicrobial agents within the microfibers and, therefore, attests to their successful immobilization.

#### 3.4.4. Thermal Stability

**Figure 4:**
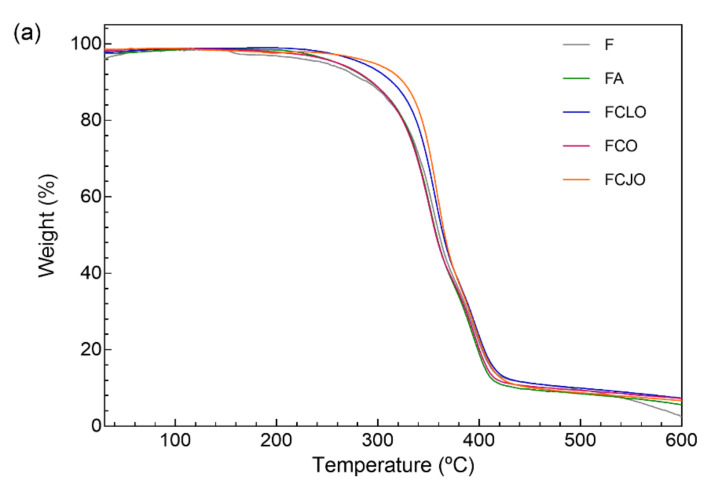

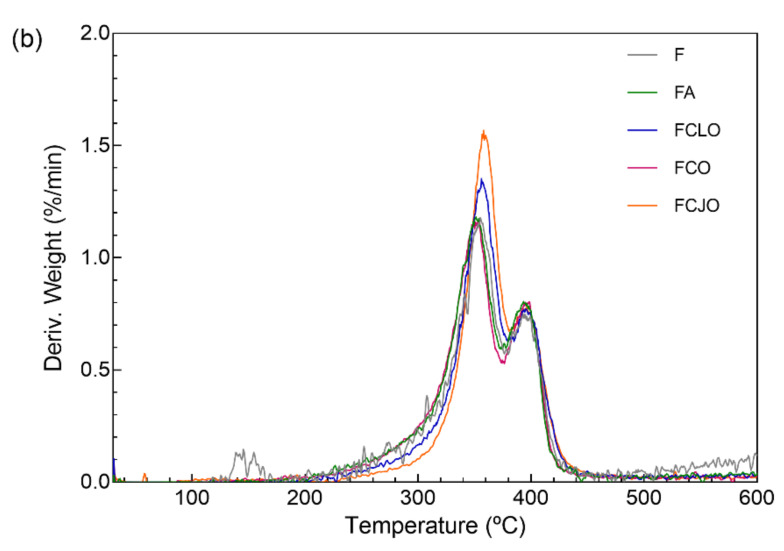
(**a**) TGA and (**b**) DTG curves of the CA/PCL microfibers, unloaded (F) and loaded with antimicrobial agents, obtained from 25 to 600 °C under nitrogen atmosphere, flow rate of 200 mL/min and temperature rise of 10 °C/min.

Degradation steps associated with temperature rise were recognized on the CA/PCL fibers via TGA and DTG (Figure 4). The first degradation step was identified at approximately 150 °C for the control samples (F) and between 220 and 240 °C for the remainder biomolecules. This indicates that the volatile nature of the EOs can be protected by being combined with polymeric matrices. Wen et al. reported just that by observing a shift in the first degradation step from ≈70 °C to ≈120 °C, when combining cinnamon oils with poly(vinyl alcohol) nanofilms [47]. Moreover, because of the reduced amount of EOs immobilized, the interactions established with the polymeric fibers may have left fewer free groups available, increasing the biomolecules’ stability and, consequently, their thermal resistance. This may also explain the differences in temperatures necessary to induce the first degradation step between the control samples (F) and the EO-treated samples. The incorporation of phenolic compounds (e.g., eugenol) within polymeric matrices has been reported to have a plasticizing effect that may shift the temperatures of degradation to higher levels [48]. This was particularly noticeable in the FCLO and FCJO. In the case of FCO, the relatively smaller diameters attained after modification (Section 3.4.2), which may have resulted from different intermolecular bonding, may also have narrowed the fiber’s thermal resistance.

From the first degradation step until the second at ≈360 °C, all samples started behaving similarly. At this point, the antimicrobial agent’s influence was neglectable. The degradation steps were then associated with the depolymerization of the CA polymeric chains, as the absorbed energy activated the undoing of the glycosidic linkages producing glucose, and to the cleavage of the PCL polymeric backbone, which initiates with the degradation of the side chains and progresses to the main chain scission [13,14,49]. The progression from the first to the second degradation steps was very smooth and gradual and represented a total mass loss of ≈88%. Interestingly, the addition of PCL to the CA polymeric solution raised the characteristic second degradation peak from ≈340 °C to ≈360 °C, thus confirming the homogeneity of the polymeric blend and the increased thermal resistance of the CA-based fibers. From 400 to 600 °C, the continuing weight loss is ascribed to further degradation of the polymers into carbon char.

### 3.5. Time-Kill Kinetics

**Figure 5:**
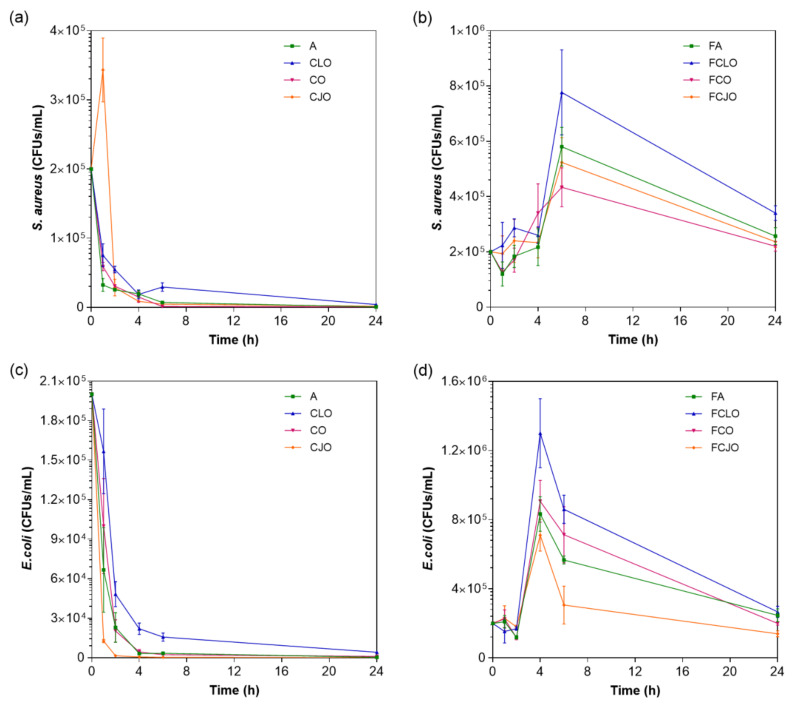
Killing-time curves of (**a**,**c**) free antimicrobial agents at microfiber loading concentration and (**b**,**d**) unloaded and loaded microfibers, against *S. aureus* and *E. coli* bacteria, up to 24 h of culture. Data derived from three repetitions. Positive controls for each bacterium (growth without agent or microfiber) were also conducted, reaching maximum values of ≈1.8 × 10^7^ and ≈2.1 × 10^7^ CFUs/mL for *S. aureus* agent-free solution and fiber-containing solution, respectively, and of ≈1.5 × 10^7^ and ≈1.1 × 10^7^ CFUs/mL for *E. coli* agent-free solution and fiber-containing solution, respectively, after 24 h culture (data not shown in graphic).

**Figure 6:**
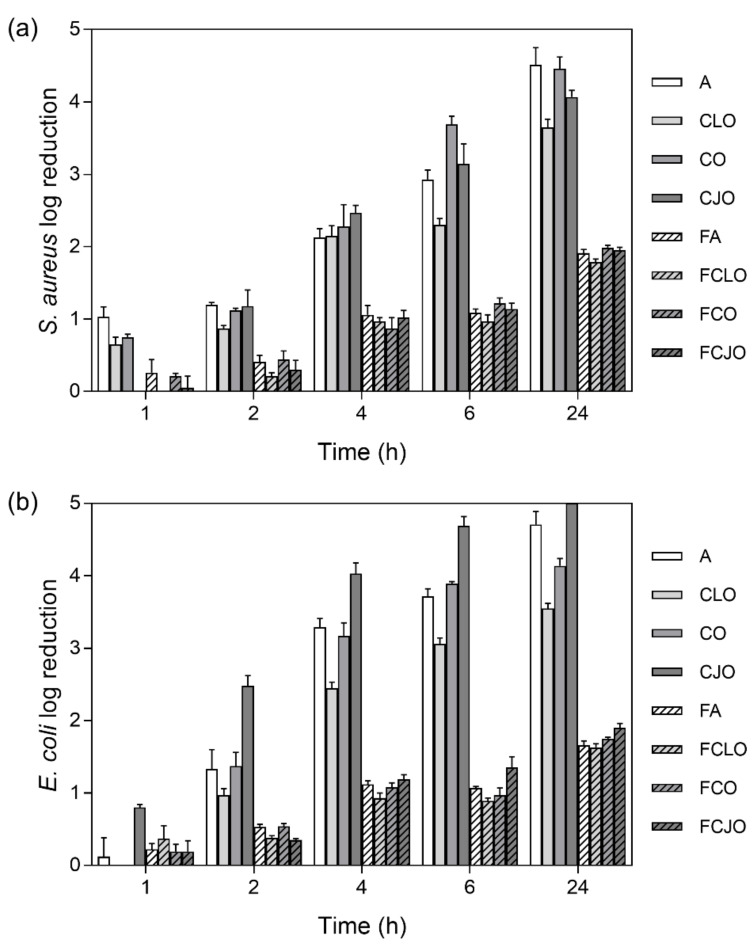
(**a**) *S. aureus* and (**b**) *E. coli* reduction (calculated as log reduction) in relation to control samples, namely bacterial inoculum without antimicrobial agents and unloaded fibers (n = 3, mean ± S.D.). The elimination of 100% of bacteria was considered as log 5.

The kill-time kinetics for each antimicrobial agent and loaded microfiber was determined by the number of remaining viable cells at specific incubation periods, 1, 2, 4, 6, and 24 h (Figure 5) and the associated log reduction (Figure 6). Data were compiled so the exposure/action time and the overall potency of the antibiotic and the EOs were established against both Gram-positive and Gram-negative bacteria free from antimicrobial agents or loaded fibers. For most of the free biomolecules, bacterial reduction was observed from the very first moments of contact. Indeed, after 2 h of incubation, ≈0.87 to ≈1.20 log reduction was verified against *S. aureus* and ≈0.97 to ≈2.48 log for *E. coli*, which represented an average decrease of ≈90% and ≈97% in the total number of viable cells, respectively. The continuous and progressive reduction in the number of viable bacteria and an increase in the log reduction values demonstrated the free agents’ effectiveness throughout time. Yet, there were no verifiable susceptibility patterns for each bacterium. For instance, CJO, which was present at the highest concentration, did not exert the quickest effect on the bacteria; in fact, in the first moments of interaction, CJO allowed *S. aureus* to grow. This inability to predict the behavior of an EO by its working concentration has been previously reported [5]. The only exception was CLO. Its bacterial response was the slowest from the group, which was proportional to the concentration in test. Regardless, after 24 h of incubation, bacteria treated with CLO experienced a reduction levels of ≈3.65 log for *S. aureus* and ≈3.55 log for *E. coli*, which were quite close to those registered by the other EOs. CLO may rely on the properties of its major component, eugenol, to affect the cells’ intracellular functions or ion transport, this way preventing important metabolites in the bacteria from working at full capacity and, ultimately, leading to the cell death [32]. Similar observations were made on the modified microfibers. Once again, FCLO displayed the lowest reduction levels, allowing bacteria to grow in the first 6 h. After that point, its action became more effective. It is likely that the intermolecular bonds generated during the 72 h immobilization period required more time to break, and consequently to allow the release of the EOs. The same growing patterns were observed for the other agents. However, their effectiveness in killing bacteria was superior to that of FCLO (Figure 6), as expected. There is yet to be a consensus on the period at which EOs are the most effective. However, it is generally accepted that they stimulate cell autolysis in exponential and stationary cell phases [50]. Our findings demonstrated that exponentially growing cells are very susceptible to the EOs’ action when in a free state, and that when immobilized their action tends to occur in a more stationary stage. Undeniably, log reduction was most significant after 24 h of culture. At this point, it was evident that *S. aureus* was more susceptible to the prolonged action of the EOs than the *E. coli*, the only exception being the CJO which was seen to act more effectively against the Gram-negative bacteria (Table 3).

### 3.6. Cell Membrane Permeability

The effect of the EOs on the Gram-positive and Gram-negative cell membrane permeability was evaluated through the percentage of REC (Table 4), after 6 h of incubation. Data reported an increase in the solution’s conductivity upon incubation with the antimicrobial agents, which indicated the cell membranes became permeable, at different levels, after treatment with the free biomolecules or the loaded fibers. This was particularly noticeable on the *S. aureus* bacteria suggesting the release of electrolytes from within the cells by cellular leakage. Indeed, the lysis and death of the bacteria by the action of ampicillin or the EOs may have lead the bacteria to release the intracellular content and with electrolytes such as K^+^, Ca^2+^, and Na^+^ capable of raising the conductivity of the solution [51]. Considering the EOs primary mechanism of action against bacteria resorts to the accumulation of biomolecules at their surface, disturbing its structure and functionality, it is likely the content leakage to be the result of their interaction with the cytoplasmic membrane [25]. The cytoplasmic membrane works as a permeability block for ions that are crucial to the cell membrane functions, namely, to maintain enzyme activity and to sustain the normal cell metabolism. Thus, regulation of ion hemostasis is of great importance to maintain the energy status of the cell. Even small variations in the membrane structure can hurtfully affect cell metabolism and lead to cell lysis [52]. This data is consistent with the SEM observations made in Figure 1, in which intracellular content release by CLO action was particularly noticeable on *S. aureus*. Overall, *S. aureus* showed higher electric conductivity than *E. coli* bacteria, which is in agreement with previous reports [53]. Finally, it is important to highlight the significant effect of CLO on the bacteria membrane permeability, even at ≈14% of its MIC value. These results demonstrate the potent action of this agent and its potential for biomedical applications.

## 4. Conclusions

The antibacterial spectrum of activity of 20 EOs was analyzed in this work. Data reported the CLO, CO, and CJO oils as the most effective against the Gram-positive bacteria *S. aureus* and the Gram-negative bacteria *E. coli*. These EOs were further characterized and, then, immobilized onto CA/PCL microfibers produced via wet-spinning. Fibers displayed a uniform and homogeneous morphology with little variations in diameter being detected upon agent immobilization. FTIR and TGA data confirmed the successful fiber modification by detecting characteristic peaks of the EOs and by demonstrating the increased overall thermal stability of the polymeric blend, respectively. Even at small amounts, below MIC value, the EO-modified microfibers promoted cell death compared to the control groups (unloaded and ampicillin-modified fibers), by disrupting and permeabilizing the cell cytoplasmic membrane. Considering only ≈14% of the MIC value of CLO were immobilized onto the CA/PCL microfibers and the antimicrobial activity promoted from that, these were regarded as the most effective. Data demonstrated the potential of CA/PCL wet-spun microfibers loaded with EOs for applications in biomedicine, in which the treatment of infections is the main target. However, it should be noticed that these tests are only preliminary work, a proof-of-concept of the feasibility of adsorbing EOs onto CA/PCL wet-spun microfibers capable of being released in physiological conditions and subsequently promote bactericidal action should be attained. Future studies are required to further explore new strategies for adequate loading amount in smaller time frames, to evaluate the mechanisms of action of the immobilized agents in detail, and their effectiveness towards selected bacterium species in prolonged exposure periods, so that antibiotic replacement can be envisioned.

## Figures and Tables

**Table 1:** List of tested essential oils (EOs)s, their origin, and density.

EO	Abbreviation	Origin	Density (g/cm^3^)
Amyris	AO	*Amyris balsamifera*	0.957
Cajeput	CJO	*Melaleuca leucadendron*	0.911
Cinnamon leaf	CLO	*Cinnamomum zeylanicum*	1.049
Citronella	CIO	*Cymbopogon winterianus*	0.882
Clove	CO	*Eugenia caryophyllus*	1.056
Eucalyptus	ELO	*Eucalyptus globulus* L.	1.465
Frankincense	FO	*Boswellia carterii*	0.857
Geranium	GO	*Pelargonium graveolens*	0.895
Himalayan cedar	HCO	*Cedrus deodara Loud.*	0.935
Lavandin	LO	*Lavandula hybrida*	0.889
Lemongrass	LGO	*Cymbopogon flexuosus*	0.890
Niaouli	NO	*Melaleuca viridiflora*	0.913
Orchid	OO	*Orchidaceae*	0.856
Palmarosa	PMO	*Cymbopogon martinii var.motia*	0.884
Patchouli	PTO	*Pogostemon patchouli*	0.960
Rosemary	RO	*Rosmarinus officinalis*	0.900
Sage	SO	*Salvia officinalis*	0.915
Star anise	SAO	*Illicium verum*	0.981
Tea tree oil	TTO	*Melaleuca alternifolia*	0.895
Wintergreen	WO	*Gaultheria procumbens*	1.182

**Table 2:** Zones of inhibition (ZoI) of selected EOs against Gram-positive and Gram-negative bacteria (n = 3, mean ± S.D.). The diameter of the holes (Ø = 6 mm) was included. Listing of antimicrobial agents and respective results were done by descent order in respect to each bacterium. Ampicillin (A) was used as positive control agent.

Antimicrobial Agents	ZoI Diameter (mm)	Antimicrobial Agents	ZoI Diameter (mm)
	*S. aureus*		*E. coli*
A	28.67 ± 0.58	A	23.50 ± 0.50
RO	18.33 ± 2.08	CJO	21.33 ± 0.58
FO	17.83 ± 0.76	TTO	14.67 ± 2.08
ELO	16.67 ± 1.15	SO	13.00 ± 2.65
NO	16.33 ± 1.53	CLO	12.83 ± 0.76
CJO	15.67 ± 1.53	NO	12.67 ± 1.15
CO	15.00 ± 1.00	CO	12.33 ± 1.58
SO	14.67 ± 0.58	RO	11.33 ± 3.21
PTO	13.50 ± 0.50	LGO	10.67 ± 0.58
TTO	13.33 ± 1.53	ELO	10.00 ± 1.00
CLO	12.67 ± 0.58	FO	9.83 ± 1.26
LGO	12.17 ± 0.29	LO	9.00 ± 1.00
PMO	11.33 ± 0.58	PMO	8.00 ± 0.50
LO	11.00 ± 1.00	OO	7.33 ± 0.58
GO	9.33 ± 0.58	GO	6.83 ± 0.29
CIO	9.17 ± 0.29	AO	-
OO	8.83 ± 0.29	CIO	-
AO	8.33 ± 0.58	HCO	-
HCO	-	PTO	-
SAO	-	SAO	-
WO	-	WO	-

**Table 3:** Minimal inhibitory concentrations (MICs) of selected EOs against Gram-positive and Gram-negative bacteria (n = 3, S.D. < ± 5.0 µg/mL). Listing of antimicrobial agents and respective results were done by descent order in respect to each bacterium. Ampicillin (A) was used as positive control agent.

Antimicrobial Agents	MICs (mg/mL)	Antimicrobial Agents	MICs (mg/mL)
	*S. aureus*		*E. coli*
CLO	0.82	CLO	0.82
CO	0.83	CO	0.83
CJO	22.38	CJO	11.19
TTO	22.38	TTO	11.39
A	0.03	A	0.03
ELO	36.63	SO	45.75
LGO	44.50	GO	89.50
NO	45.65	LGO	89.00
PTO	48.00	NO	91.30
CIO	88.20	ELO	146.50
LO	88.90	RO	180.02
RO	90.01	OO	342.40
SO	91.50	FO	342.80
OO	171.20	PMO	353.60
PMO	176.80	CIO	>352.80
AO	191.40	LO	>355.60
WO	236.40	HCO	>374.00
GO	358.00	AO	>382.80
FO	>342.80	PTO	>384.00
HCO	>374.00	SAO	>392.40
SAO	>392.40	WO	>472.80

**Table 4:** Effect of the antimicrobial agents in free state and loaded onto CA/PCL microfibers, in the cell membrane permeability of *S. aureus* and *E. coli* bacteria (n = 3, S.D. < ±0.5%). Solutions containing the free agents were prepared at concentrations equal to those of microfiber loading. The bacteria inoculum without any agent (C) and the unloaded fibers (F) were used as control.

REC (%)										
Bacteria	C	A	CLO	CO	CJO	F	FA	FCLO	FCO	FCJO
*S. aureus*	−0.49	19.10	9.81	19.83	20.41	−0.29	1.96	1.10	3.19	8.34
*E. coli*	−1.01	15.50	6.08	12.69	25.09	−1.32	0.75	0.67	1.69	4.49

## Supplementary Materials

The following are available online at https://www.mdpi.com/2218-273X/10/8/1129/s1, Figure S1: Schematic representation of the employed wet-spinning apparatus, Table S1: ZoI of selected EOs against Gram-positive and Gram-negative bacteria. Images were collected without regard for size proportionality, being only used as representations of the halos formed. Listing of antimicrobial agents was organized by alphabetical order. Ampicillin (A) was used as positive control agent.
