# Supplementary material for: Physical, Thermal, and Antibacterial Effects of Active Essential Oils with Potential for Biomedical Applications Loaded onto Cellulose Acetate/Polycaprolactone Wet-Spun Microfibers
Source: Biomolecules. 2020 Jul 31;10(8):1129. doi: 10.3390/biom10081129 (PMC7465996; doi:10.3390/biom10081129)
Supplement: Supplementary file 1 [file biomolecules-10-01129-s001.pdf]

biomolecules

Supporting Information

Article

Physical, Thermal, and Antibacterial Effects of Active Essential Oils with Potential for Biomedical Applications Loaded onto Cellulose Acetate/Polycaprolactone Wet-Spun Microfibers

Helena P. Felgueiras *, Natália C. Homem, Marta A. Teixeira, Ana R. M. Ribeiro, Joana C. Antunes and Maria Teresa P. Amorim

Centre for Textile Science and Technology (2C2T), Department of Textile Engineering, University of Minho, Campus of Azurém, 4800-058 Guimarães, Portugal; natalia.homem@2c2t.uminho.pt (N.C.H.); martaalbertinateixeira@gmail.com (M.A.T.); rita.ribeiro_02@hotmail.com (A.R.M.R.); joana.antunes@2c2t.uminho.pt (J.C.A.); mtamorim@det.uminho.pt (M.T.P.A.)

* Correspondence: helena.felgueiras@2c2t.uminho.pt; Tel.: +351-253-510-283; Fax: +351-253-510-293

Received: 29 June 2020; Accepted: 30 July 2020; Published: 31 July 2020

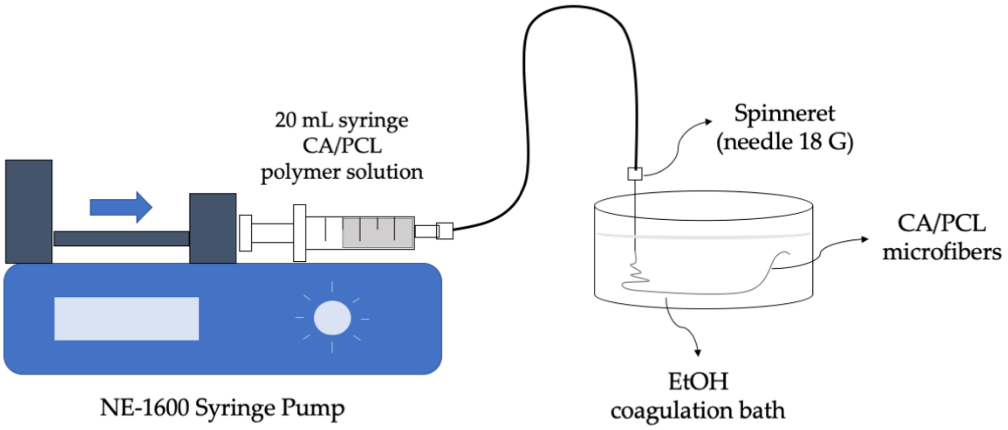

Figure 1. Schematic representation of the employed wet-spinning apparatus.

Table 1. ZoI of selected EOs against Gram-positive and Gram-negative bacteria. Images were collected without regard for size proportionality, being only used as representations of the halos formed. Listing of antimicrobial agents was organized by alphabetical order. Ampicillin (A) was used as positive control agent.

**Antimicrobial Agents**	**ZoI Diameter (mm)**	
	S. aureus	***E. coli***
A	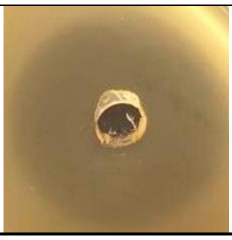	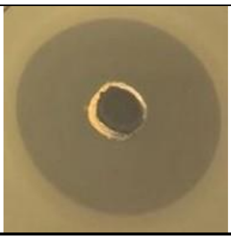

AO	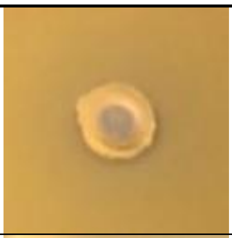	-
**CJO**	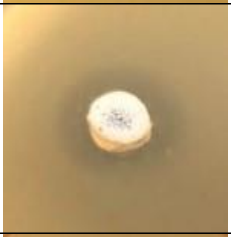	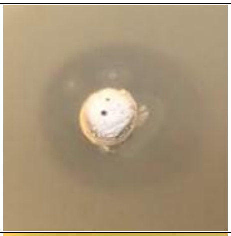
**CLO**	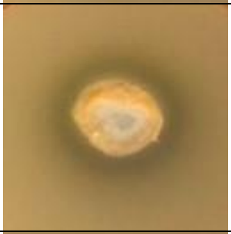	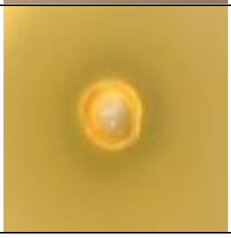
CIO	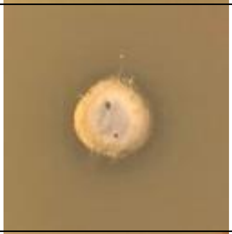	-
**CO**	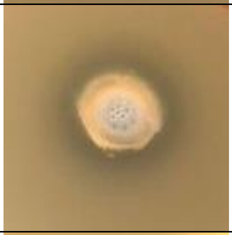	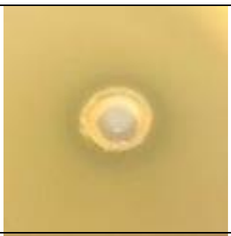
ELO	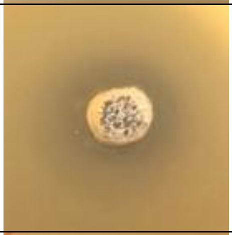	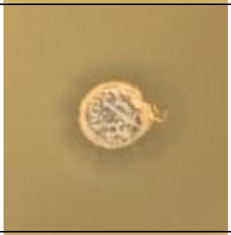
FO	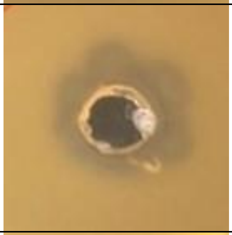	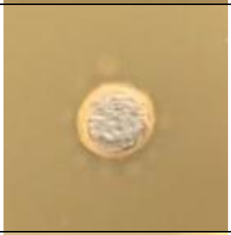
GO	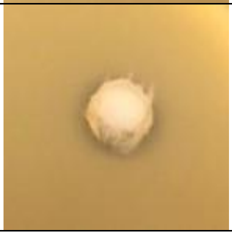	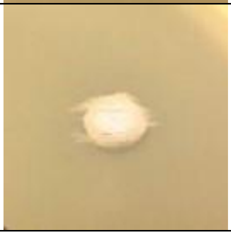
HCO	-	-

LO	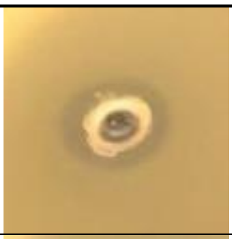	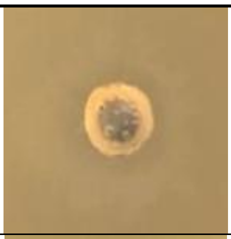
LGO	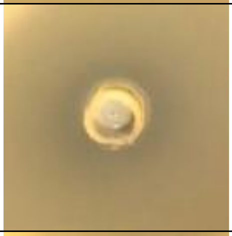	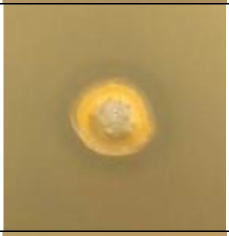
NO	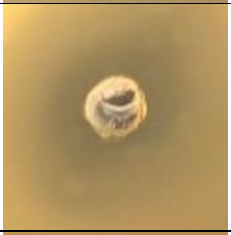	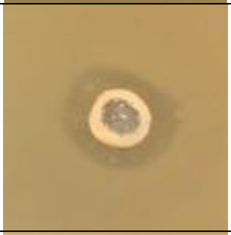
OO	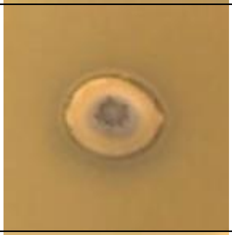	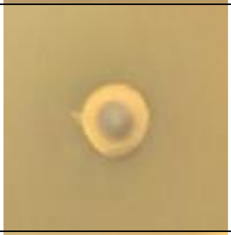
PMO	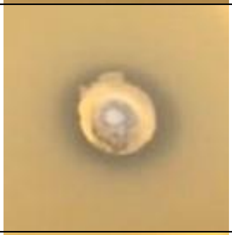	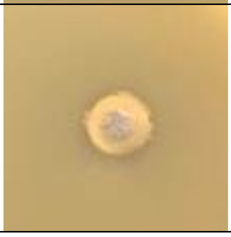
PTO	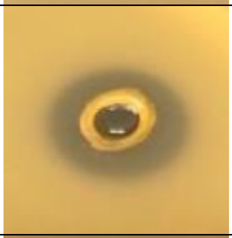	-
RO	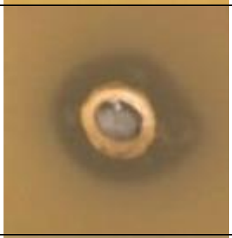	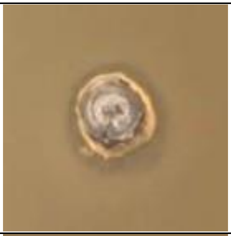
SO	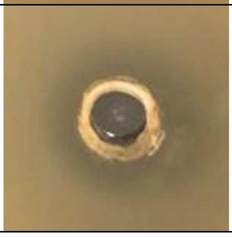	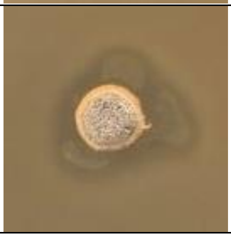
SAO	-	-

TTO	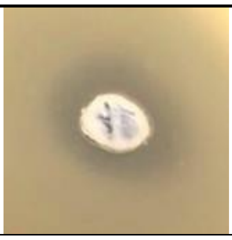	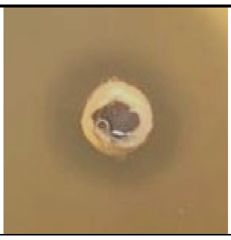
WO	-	-

© 2020 by the authors. Submitted for possible open access publication under the terms and conditions of the Creative Commons Attribution (CC BY) license (http://creativecommons.org/licenses/by/4.0/).
